# Comparative study of antimicrobial action of aloe vera and antibiotics against different bacterial isolates from skin infection

**DOI:** 10.1002/vms3.488

**Published:** 2021-05-05

**Authors:** Safia Arbab, Hanif Ullah, Wang Weiwei, Xiaojuan Wei, Salah Uddin Ahmad, Lingyu Wu, Jiyu Zhang

**Affiliations:** ^1^ Key Laboratory of Veterinary Pharmaceutical Development Ministry of Agriculture Lanzhou China; ^2^ Lanzhou Institute of Husbandry and Pharmaceutical Sciences Chinese Academy of Agricultural Sciences Lanzhou China; ^3^ Key Laboratory of New Animal Drug Project of Gansu Province Lanzhou China; ^4^ Key Laboratory of Animal Parasitology of Ministry of Agriculture and Rural Affairs Shanghai Veterinary Research Institute Chinese Academy of Agricultural Sciences Shanghai China

**Keywords:** aloe vera, antibacterial, extract, skin infection, zone inhibition

## Abstract

Aloe vera is reputed to have medicinal properties. For centuries, it has been used for an array of ailments such as mild fever, wounds and burns, gastrointestinal disorders, diabetes, sexual vitality and fertility problems to cancer, immune modulation, AIDS and various skin infections. In present study, antibacterial activity of aloe vera gel extracts was tested against some common skin infection pathogens, that is, *Escherichia coli*, *Shigella, Salmonella* spp. and *Staphylococcus aureus* all were recorded positive. Antibiotic resistance and susceptibility pattern of above isolates were also studied against 10 clinically significant antibiotics (ampicillin [AMC], amoxicillin, augmentin, cefotaxime, ceftazidime [CAZ], cefuroxime [CXM], ciprofloxaci, tetracycline, cefpodoxime and imipenem). AMC and CXM were found to be most effective antibiotic followed by CXM with highest efficacy against Gram‐negative bacteria. In case of CAZ showed highest efficacy was showed against Gram‐positive bacteria. Aloe vera leave gel was extracted with four different solvent‐like aloe vera leaf extract, root extract, leaf ethanol extract and root ethanol extract; however, Gram‐negative as well Gram‐positive isolates was found highest susceptibility with aloe leaf and aloe root ethanol extract. Moderate sensitivity observed with aloe leaf extract and aloe root extract against both Gram‐positive as well as Gram‐negative bacterial isolates. This result showed that ethanol extracts of aloe vera both leaf and root can be used alongside conventional antibiotics to fight agents of infections that are so prevalent in the skin infection.

## 1 INTRODUCTION

In recent years, multiple drug resistance in human pathogenic microorganisms has developed due to indiscriminate usage of commercial antimicrobial drugs for the treatment of infectious diseases. This scenario forced scientists for searching new antimicrobial products from distinct sources, like medicinal plants, which are the better sources of novel antimicrobial chemotherapeutic agents. Infectious diseases are concerned to be pursued in majority of health institutions, pharmaceutical companies and governments all over the world (accounting for over 50,000 deaths every day), especially with the current raising trends of multidrug resistance among emerging and reemerging bacterial pathogens to the available modern drugs or antibiotics. The search for newer sources of antibiotics is a global challenge in preoccupying research institutions, pharmaceutical companies and academia, since many infectious agents are becoming resistant to synthetic drugs. It is therefore very necessary that the process of searching newer antibiotic sources. Plants are the cheapest and safer alternative sources of antimicrobials (Kumar et al., 2012).

Aloe vera (Aloe barbadensis miller) is a plant, which belongs to the family of Liliaceae and is mostly succulent with a whorl of elongated, pointed leaves (Muir‐Beckford & Badrie, 2000; Strickland et al., 2004). The name is derived from the Arabic word ‘alloeh’ which means ‘bitter’, referring to the taste of the liquid contained in the leaves. The term ALOE refers to a solid residue obtained by evaporating the latex derived from the outer layers of the plant leaf. Taxonomists now refer to aloe barbadensis as aloe vera. The central bulk of the leaf contains colorless mucilaginous pulp, made up of large, thin‐walled mesophyll cells containing the aloe vera gel itself. Despite its wide use as a folk remedy over a long period of time, the biochemical details of its action on physiologica pathophysiological functions have not been systematically investigated (Rajasekaran et al., 2006).

Medicinal plants of the lily family (Liliaceae), genus Aloe, have been used for the treatment of skin diseases for more than 5,000 years. Among more than 360 Aloe species, aloe vera (Aloe barbadensis miller) has been the most popular in both folk and officinal medicine (Millikan, 2003). Aloe vera extracts are widely used in a variety of over‐the‐counter and dermatological products. Many studies report the effective use of this plant when applied topically for the treatment of burns, sunburns, inflammatory skin disorders and wounds (Dal'Belo et al., 2006).

Bacterial resistance to antibiotics is increasingly becoming a concern to animal health. Currently used antibiotic agents are failing to end many bacterial infections due to super resistant strains. For this reason, the search is ongoing for new antimicrobial agents, either by the design and synthesis of new agents or through the search of natural sources for as yet undiscovered antimicrobial agents. Herbal medications in particular have seen a revival of interest due to a perception that there is a lower incidence of adverse reactions to plant preparations compared to synthetic pharmaceuticals. Coupled with the reduced costs of plant preparations, this makes the search for natural therapeutics an attractive option.

## 2 MATERIAL AND METHODS

### 2.1 Samples collection

Skin infection isolates were obtained from septic wounds at different animal. Wound exudates were obtained from the infected sites of each animal with sterile cotton swabs and applied to freshly prepared slants of nutrient agar and Mannitol Salt agar (Oxoid). The cultures were then transferred to the laboratory where they were incubated at 37°C for 24 hr (Huys et al., 2002).

### 2.2 Bacterial isolates, culture media and species identification

Colonies growing on slants were streaked on top of freshly prepared plates of Mannitol Salt agar and Brain Heart Infusion agar and incubated again. Primary characterization of isolates was based on the Gram stain, morphological and cultural characteristics. Identification also includes growth on different media including Nutrient agar, nutrient broth at 37°C for the determination of microbial growth and then subcultured on to blood agar, chocolate agar, Sorbitol Macconkey Agar, Eosin Methylene Blue Agar, and *Salmonella* agar plates incubated at 37°C for 24 hr (Oxoid). Catalase and coagulase tests were also performed for biochemical characterization (Udo et al., 2006).

### 2.3 Maintenance of clinical isolates

Stock cultures were maintained in vials by growing the skin isolates in 3‐ml nutrient broth and next day overlaying with 3 ml 40% glycerol. Vials were than frozen at −70°C (Richardson et al., 2005).

### 2.4 Determination of antibiotic resistance profile

Skin wound isolates were subjected to antibiotic resistance screening by disk diffusion method. For this purpose, inoculates were prepared by diluting overnight cultures in sterile sodium chloride (0.9%) suspension and then match with the 0.5 standard Mac Farland index. Bacterial suspensions were then plated onto Mueller‐Hinton agar (Oxoid) and the commercially available antibiotic discs were placed on lawn of culture and the plates were incubated over night at 37°C (Kos et al., 2006). Sensitivity, intermediate sensitivity and resistances were determined by the zone of complete growth inhibition around each disk according to reference standards. The following antibiotic discs were used ampicillin (AMC) (10 μg), amoxicillin (AMX) (25 μg), augmentin (AUG) (30 μg), cefotaxime (CTX) (30 μg), ceftazidime (CAZ) (30 μg), cefuroxime (CXM) (30 μg), ciprofloxacin (CPX) (10 μg), tetracycline (30 μg), cefpodoxime (CP) (10 μg) and imipenem (10 μg).

### 2.5 Preparation of aloe vera gel and extracts

Aloe vera plants were purchased from a nursery. The gel was taken from the leaves into a clean container and used as such (Agarry et al., 2005). While the leaves from which the gel has been drained were air dried (50 g), macerated with 100‐ml sterile distilled water in a warning blender for 10 min. The macerate was first filtered through doubled layered muslin cloth and then centrifuged at 4,000 *g* for 30 min. The supernatant fluid was filtered through Wattman No. 1 filter paper and heat sterilized. The extract was preserved aseptically in a brown bottle at 5°C until used (Sayaka and Watanabe, 2003).

### 2.6 Antimicrobial susceptibility testing of aloe vera

Sterile agar (at 45°C) was poured into sterile Petri dishes, which had been inoculated with the test organisms. The plates were allowed to gel for an hour. Wells (10‐mm diameter) were made with the aid of flamed cork borer on the surface of the agar plates. About 0.1 ml of each of the gel and the leaf extracts were delivered into each of the wells. These were incubated at 37°C for 24 hr. The presence of zones of inhibition was regarded as the presence of.

### 2.7 Statistical analysis

The graphic representation was performed using the program (Microsoft Office Excel, 2007).

## 3 RESULTS

### 3.1 Confirmation of bacterial isolates

The overall percentage prevalence of positive bacteria isolates from skin wounds of animal. A total of 150 wound samples were examined and all were recorded positive for different organisms. The percentage prevalence of organisms is presented in (Table 1). Out of 150 samples, 55, 35, 10 and 40 were found positive for *Escherichia coli*, *Shigella*, *Salmonella* spp. and *Staphylococcus aureus*, respectively. All organisms were identified on their morphological, cultural characteristics and staining reactions. Organisms were further confirmed by their biochemical reactions.

**TABLE 1:** The number and percentage prevalence of bacterial organisms isolated from skin infection of animal

Isolates	Total number of organisms	Total percentage
Gram–negative	100	
*Escherichia coli*	55	36.66%
*Shigella* spp.	35	23.33%
*Salmonella* spp.	10	6.66%
Gram‐positive	50	
*Staphylococcus* aureus	40	26.66%

### 3.2 Susceptibility of isolated organisms against different extract of aloe vera

**FIGURE 1:**
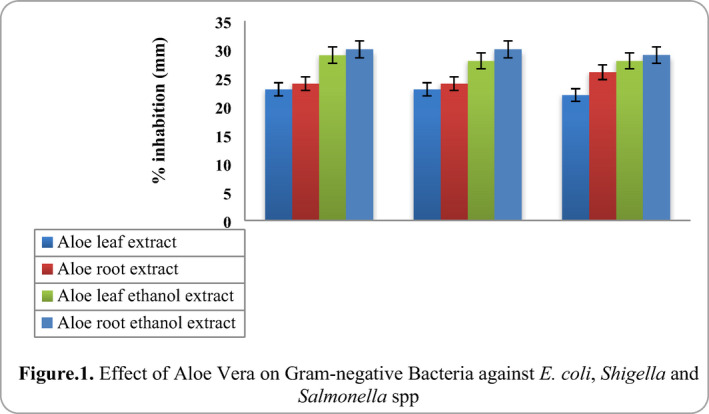
Effect of aloe vera on Gram‐negative bacteria against *Escherichia coli*, *Shigella* and *Salmonella spp*

In our study, in vitro antimicrobial properties of aloe vera gel 100 µl were investigated against various common pathogenic bacteria. The well diffusion method showed significant zone of inhibition against all the pathogens tested, and the results are comparable to the conventional antibiotics. The antimicrobial activity of the extracts and their potency was quantitatively assessed by the presence of inhibition zone and zone diameter. Result indicated that 100 µl of leaf extract of aloe vera and root extract of aloe vera produced a clear zone and organisms showed quite sensitive reaction was formed around the disc, whereas aloe leaf ethanol extract and aloe root ethanol extract showed greater susceptibility against gram‐negative bacteria isolates and the results are given in Figure 1.

**FIGURE 2:**
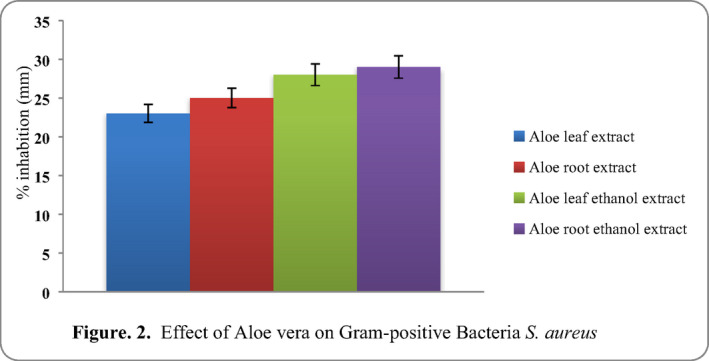
Effect of aloe vera on Gram‐positive bacteria *Staphylococcus aureus*

*Staphylococcus aureus* had the quite zone of inhibition diameter of on both aloe leaf and root extract respectively at 100 µl, whereas the ethanol extract had significant effect on the zone of inhibition of *S. aureus*. At 100 µl of the ethanol extract, the highest zone of inhibition of (aloe leaf ethanol and aloe root ethanol, susceptibility against gram‐positive bacteria isolates and the results are given in Figure 2).

### 3.3 Susceptibility of isolated organisms against different antibiotics

**FIGURE 3:**
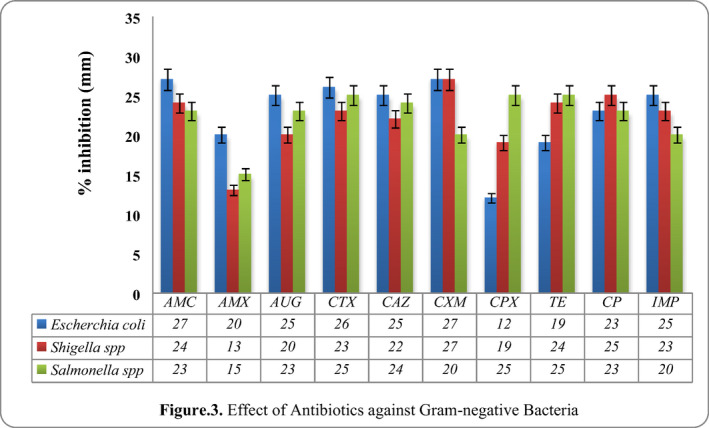
Effect of antibiotics against Gram‐negative bacteria

In present study, it was found that the effect of antibiotics for gram‐negative as well as gram‐positive. The effect of antibiotics showed greater susceptibility followed by (AMC, CXM, CTX, AUG, CAZ and imipenem) on *E. *coli, *Shigella* spp. (imipenem, CXM, CP, tetracycline and AMC) and *Salmonella* spp. (CTX, CPX, tetracycline and CAZ) respectively, and the results are presented in Figure 3.

**FIGURE 4:**
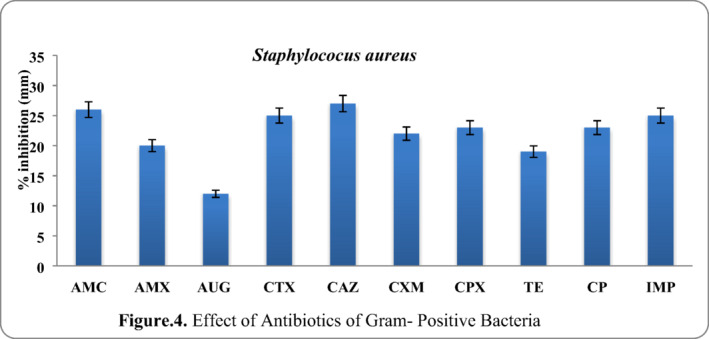
Effect of antibiotics of Gram‐positive bacteria. AMC, ampicillin; AMX, amoxicillin; AUG, augmentin; CAZ, ceftazidime; CP, cefpodoxime; CPX, ciprofloxacin; CTX, cefotaxime; CXM, cefuroxime; IPM, imipenem; TE, tetracycline

Whereas (ceftazidme, AMC, CTX and imipenem) produced maximum susceptibility for *S*. *aureus*, antibiotics showed susceptibility against gram isolate and the results are given in Figure 4.

### 3.4 Comparison of antibacterial activity of different extract of aloe vera with standard antibiotics against Gram‐negative and Gram‐positive bacteria

**FIGURE 5:**
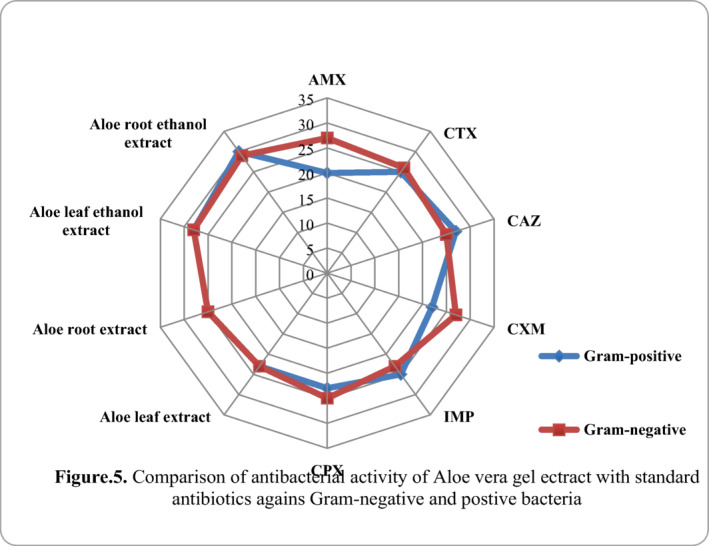
Comparison of antibacterial activity of aloe vera gel extract with standard antibiotics against Gram‐negative and Gram‐positive bacteria

It was found that at 100 µl concentration of aloe vera leaf and root extract showed similar sensitivity as that of standard antibiotics used against all bacterial isolates Gram‐negative as well as Gram‐positive, respectively. Whereas the antibiotic activity at 100 µl of ethanol leaf and ethanol root extract of aloe vera showed greater susceptibility against all organisms isolated, during in this study is somewhat exhibited by some standard antibiotics showed less susceptibility. Hence, it is proved that different extract of ethanol extracted aloe vera produced showed highest activity as compare to standard antibiotics and the results are given in Figure 5.

## 4 DISCUSSION

Aloe vera (Aloe barbadensis miller) is a cactus‐like xerophytes plant, and about 360 species have been identified. It is used to treat various skin problems (skin irritation, burns, wound, acne and dermatitis) and has also been used to heal skin exposure to ultra violet and gamma radiation (Zhang et al., 2006). This plant is recognized to have medicinal properties and has been used for wide variety of ailments such as mild fever, wounds and burns, gastrointestinal disorders, diabetes, sexual vitality, fertility problems, cancer, immune modulation, AIDS and various skin diseases (Chatterjee et al., 2015). Numerous studies report the effectual use of this plant when functioning topically for the healing of burns, sunburns, inflammatory skin disorders and wounds (Dal'Belo et al., 2006).

Four bacterial species were recognized from skin wound samples, *S. aureus*, *E. coli*, *Salmonella* and *Shigella* spp. which were the most prevalent bacteria encountered in skin wounds. This is reported that *E. coli*, *S. aureus*, and *Shigella and Salmonella spp*., their incidence percentages recorded were 36.66%, 26.66%, 23.33%, and 6.66%. In a similar manner, various workers throughout world did high rate to isolate the bacterial organisms from wound samples of animals. However, the comparison of the present figures can be compared with the results of (Arbab et al., 2021; Rind & Khan, 2000).

The large sizes of zones growth inhibition produced by aloe extracts and the 10 antibiotics (AMC, AMX, AUG, CTX, CAZ, CXM, CPX, tetracycline, CP and imipenem) against the four bacterial *E. *coli, *Shigella* spp. *Salmonella* spp. and *S. aureus* indicated the potency of the active constituents in aloe vera and those antibiotics. The aloe root ethanol extract showed the greatest effect on both all isolates organisms compared to the leaf and root water extract and the leaf ethanol extract. This is an indication that aloe is effective against *S. aureus Staphylococcus epidermidis*, *Pseudomonas aeruginosa* and *S*. *pyogene* infections. The phytoconstituents of aloe have longed been known as its antibacterial properties have been widely reported (Arbab et al., 2020; Bashir et al., 2011). However, most reports on the activity of aloe have focused mainly on the commensal micro flora and community acquired infections, while information is on its activity against animal‐based pathogens is scanty.

Another researcher study conducted to check the antibacterial activity of ethanol and chloroform extract of Aloe Vera, ethanol extract of aloe vera exhibited maximum inhibition against *S. aureus*, *S*. *pneumonia* and *B*. *subtilis* (Jothi et al., 2014). This finding is also consistent with other reports investigation was carried out and ethanol extract showed the greatest effect on *S. aureus, E. coli, Klebisella pneumonia* and *Shigella* compared to pure aloe extract. Using ethanol extracts the zones of inhibition (Rudrangshu et al., 2015).

The current study also showed agreement with the previous studies (Agarry et al., 2005). The result of current study is generally the ethanol extract showed 100% efficacy and susceptibility against Gram‐positive as well as Gram‐negative isolates showed greater result as compare to crude extract because ethanol's are more polar than water (Matu & Van Staden, 2003). A similar kind of investigation was carried out and ethanol extract showed the greatest effect on *S. aureus, E. coli, Klebisella pneumonia* and *shigella* (Rudrangshu et al., 2015).

Aloe vera is a promising plant material with numerous biological activities. Various solvents were used for extraction of bioactive compound from aloe and the extract yields were measured. Highest percentage yield obtained with ethanol followed by water. Antimicrobial activity of these extract was tested with two different pathogenic bacteria by disc method. The results of this testing shed light into the antimicrobial abilities of test substance, potentially providing ground for natural alternatives to pharmaceutical antibiotics medication. This study has consistently demonstrated the effectiveness of aloe as an antibacterial agent against gram‐negative and as well as gram‐positive bacteria whereas the 10 antibiotics tested also showed the ability to inhibit the growth of bacterial pathogen. Aloe vera can be used for the development of broad spectrum antibiotics.

## 5 CONCLUSION

The present study has imparted that the ethanol leaf and root extracts of aloe vera gel has intended effect of antibacterial activity against both Gram‐positive and as well as Gram‐negative bacteria. This investigation further assures that the plant extracts could be used for the treatment of microbial infections. Hence, our present findings will be recommended that aloe vera gel at optimum concentration could be used as an antiseptic for prevention of some microbial skin wound infections. It is believed that this study could be used to identify new and more potent antimicrobial drugs of natural origin.

## CONFLICTS OF INTEREST

The authors declare that there are no conflicts of interest.

## AUTHOR CONTRIBUTION

**Hanif Ullah:** Conceptualization; Formal analysis; Writing‐review & editing. **ji yu zhang:** Funding acquisition; Investigation; Project administration; Supervision. **weiwei Wang:** Writing‐review & editing. **Xiaojuan Wei:** Visualization. **Lingyu Wu:** Writing‐review & editing. **Salah Uddin Ahmad:** Writing‐review & editing.

## ETHICAL STATEMENT

All animal experiment were conducted according to the guidelines for animal use in toxicology with under the research topic of ‘Comparative Studies of Antimicrobial Action of Aloe Vera and Antibiotics against Different Bacterial Isolates from Skin infection’. All experiments were approved by the Animal Administration and Ethics Committee of Lanzhou Institute of Husbandry and Pharmaceutical Sciences, Chinese Academy of Agricultural Sciences. The certificate number was SCXK (Gan) 2019–001.

### PEER REVIEW

The peer review history for this article is available at https://publons.com/publon/10.1002/vms3.488.

## Data Availability

The data that support the findings of this study are openly available in repository.
